# Avoidance learning: a review of theoretical models and recent developments

**DOI:** 10.3389/fnbeh.2015.00189

**Published:** 2015-07-21

**Authors:** Angelos-Miltiadis Krypotos, Marieke Effting, Merel Kindt, Tom Beckers

**Affiliations:** ^1^Department of Clinical Psychology, University of AmsterdamAmsterdam, Netherlands; ^2^Amsterdam Brain and Cognition, University of AmsterdamAmsterdam, Netherlands; ^3^Department of Psychology, KU LeuvenLeuven, Belgium

**Keywords:** avoidance, fear, anxiety, learning, neuroscience

## Abstract

Avoidance is a key characteristic of adaptive and maladaptive fear. Here, we review past and contemporary theories of avoidance learning. Based on the theories, experimental findings and clinical observations reviewed, we distill key principles of how adaptive and maladaptive avoidance behavior is acquired and maintained. We highlight clinical implications of avoidance learning theories and describe intervention strategies that could reduce maladaptive avoidance and prevent its return. We end with a brief overview of recent developments and avenues for further research.

## Introduction

Avoidance of genuinely threatening stimuli or situations is a key characteristic of adaptive fear. People will typically not enter a building after a major earthquake nor approach a stray lion. At the same time, excessive avoidance in the absence of real threat can severely impair individuals' quality of life and may stop them from encountering anxiety-correcting information (Barlow, 2002). In such cases, avoidance loses its adaptive value and may transform into a maladaptive response. Maladaptive avoidance is in fact a central characteristic of a wide spectrum of mental disorders (World Health Organization, 2004; American Psychiatric Association, 2013). Individuals with Obsessive Compulsive Disorder (OCD), for instance, tend to avoid situations in which the potential for contact with contaminants is high (Rachman, 2004), Post-Traumatic Stress Disorder (PTSD) patients will try to avoid intrusive memories (Brewin and Holmes, 2003; Williams and Moulds, 2007), and social phobics will refuse to attend group gatherings (Bögels et al., 2010; Schneier et al., 2011).

**Figure 1:**
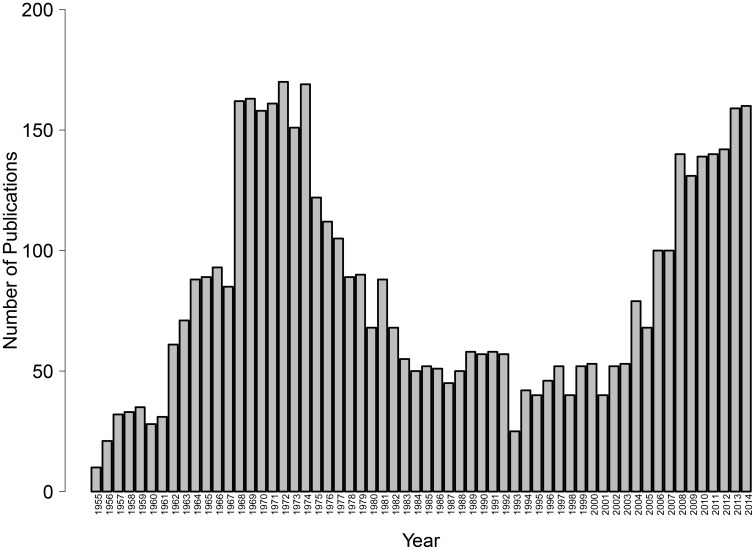
**Number of publications indexed in Thomson Reuters' Web of Science, research area psychology, that have the word “avoidance” in their title, by year, 1955–2014 (as of May 12, 2015)**.

Given the key role of avoidance in normal and disordered psychological functioning, it is critical to better understand the relevant conditions and psychological mechanisms responsible for the learning of avoidant reactions. Alas, although avoidance learning was once a central topic in basic psychological research, interest has waned since the 1970's, leaving important questions unanswered. Only recently has there been a resurgence of theoretical, experimental and clinical interest in the study of avoidance (see Figure 1). In the last years, new psychological theories of avoidance learning have been proposed (e.g., De Houwer et al., 2005; Lovibond, 2006) and avoidance is quickly becoming a topic of prime empirical interest not only in experimental psychology but also in clinical psychology and psychiatry as well as in behavioral neuroscience (see the present special issue). The latest edition of the Diagnostic and Statistical Manual of Mental Disorders (DSM-5; American Psychiatric Association, 2013) includes avoidance in several diagnostic criteria that previously referred to fear only. In parallel, recent years have brought rapid increases in our understanding of the brain processes involved in the learning (e.g., Delgado et al., 2009), expression (e.g., Cominski et al., 2014), and reduction (e.g., McCue et al., 2014) of avoidance behavior.

In this paper we review the main historical and modern theories of avoidance learning and present a set of principles of avoidance learning that integrate those theoretical propositions with the strongest experimental support. We also address the clinical implications of those principles and relate them to current and novel interventions for maladaptive avoidance such as in anxiety disorders or PTSD. Lastly, we consider recent findings from behavioral neuroscience.

The outline of the paper is as follows: We first describe how avoidance learning is studied in laboratory settings and how functionally similar behaviors can serve the *avoidance of* or the *escape from* an aversive event. Next, we discuss traditional theories of avoidance learning, including Mowrer's two-factor theory. In the third section we describe Bolles' (1970, 1971) Species-Specific Defense Reactions (SSDR) theory. We then review more recent theories of avoidance learning that address informational factors (e.g., expectancies) in avoidance. Next, we propose a set of principles for avoidance learning that incorporates the most well-validated propositions of the aforementioned theories. We end our review with suggestions for closer alignment between basic and clinical science and a few avenues for future research.

## Laboratory procedures for studying avoidance learning

Avoidance learning procedures typically entail the cancelation of an impending aversive event by either the emission or inhibition of an experimenter-designated response. In *active avoidance procedures*, for example, an antecedent stimulus is followed by an aversive event *unless* an experimenter-designated response is executed, a response that typically also terminates the antecedent stimulus. For example, dogs will learn to jump a barrier following the presentation of a light, previously associated with shock administration (Solomon and Wynne, 1953).

By contrast, in *passive avoidance procedures*, the aversive event occurs only if an experimenter-designated response is executed during the antecedent stimulus presentation. For example, in a standard passive avoidance procedure for rats, a rat is placed in a brightly lit compartment of a two-compartment box, with the second compartment being dark and the two compartments separated by a closed door (Venable and Kelly, 1990; Kaminsky et al., 2001). Given that rats have a preference for dark compared to lit environments (see Costall et al., 1989; Bourin and Hascoët, 2003), they will move to the dark compartment once the door is opened, an action that will be followed by shock administration. This procedure often results in the rats passively avoiding the shock by remaining in the light compartment on future occasions.

In avoidance procedures, the experimenter-designated response is not necessarily performed prior to the aversive outcome, but can also be performed in presence of it. In such cases, it would be more accurate to categorize the performed response as escape rather than avoidance. Escape responses involve distancing oneself from an *ongoing* aversive event while avoidance refers to behavior that causes the omission of a *forthcoming* noxious outcome, predicted by an antecedent stimulus (Bowrer and Hilgard, 1981; see also the distinction between antecedent events and aversive outcomes in Lovibond and Rapee, 1993). Thus, what differentiates avoidance from escape is the proximity of threat (imminent vs. ongoing).

An elegant animal laboratory procedure to differentiate between avoidance and escape responses, especially relevant to behavioral neuroscience, is the elevated T-maze task (ETM; Pellow et al., 1985). The ETM has been primarily designed for testing rats' defensive reactions in innately fearful environments (i.e., open spaces and heights; Montgomery, 1955). It typically consists of three elevated arms with one arm surrounded by a wall (enclosed arm) and the other two being open (open arms). Initially, the rat is placed in the enclosed arm. While exploring the rat will eventually end up in the open arms. Following repeated trials, the rat will tend to remain longer in the enclosed arms after being placed there (i.e., passive avoidance) or run toward the enclosed arm after being placed in one of the open arms (i.e., escape).

By using this procedure, research has illuminated the differences in the neurobiology of avoidance and escape. Specifically, serotonin, an anxiogenic neurotransmitter relevant for defensive responses (Graeff, 2002), seems to play a different role in the two types of behaviors (Zangrossi et al., 2001), with serotonin administration enhancing avoidance and inhibiting escape (Graeff, 1991). That observation supports the argument that avoidance and escape may constitute diverse types of defensive behaviors, differently elicited as a function of the proximity of threat (imminent or ongoing), a hypothesis also in line with models associating defensive response selection to predatory imminence (Fanselow, 1994). Of note, the difference between escape and avoidance is also relevant for clinical practice. Specifically, Deakin and Graeff (1991) suggested that (passive) avoidance is mainly related to generalized anxiety disorder (GAD), where a threatening event is typically anticipated, and escape to panic disorder, where panic reactions could be considered as responses to an ongoing perceived danger (Shuhama et al., 2007). This suggestion has gathered partial support from pharmacological studies. It has been demonstrated that commonly prescribed anxiolytic drugs (e.g., diazepam) result in passive avoidance deficiencies while leaving escape behavior intact. On the contrary, cholecystokinin agonists, which typically invoke panic attacks, facilitate escape behaviors (Pinheiro et al., 2007; Graeff and Zangrossi, 2010). Taken together, avoidance and escape seem to be distinct subtypes of defensive behaviors, and that they might play a different role in mental disorders.

Active and passive avoidance and escape procedures have proved valuable for testing avoidance and/or escape learning in laboratory settings. Based on findings obtained with those procedures, theories have emerged that address the underlying psychological mechanisms. We now turn to a discussion of early theories of avoidance/escape learning in psychology.

## Early theories of avoidance/escape learning and the two-factor theory

In the early days of psychology, learned avoidance was considered an example of a Pavlovian conditioned reflex (Bekhterev, 1907, 1913; Watson, 1916). Just like Pavlov's dogs would salivate upon the sound of a metronome previously associated with food administration (Pavlov, 1927), in the studies of Bekhterev (1913), a dog would flex its leg after the presentation of an antecedent stimulus, previously associated with shock administration (Herrnstein, 1969; Bolles, 1972). Since leg flexion would occur in the presence of the antecedent stimulus and prior to shock delivery, the acquired response was considered to reflect Pavlovian learning.

Nonetheless, two procedural characteristics differentiated the acquired responses from learned Pavlovian reflexes. First, what constituted the avoidance response (e.g., leg flexion) was usually an experimenter-defined voluntary response, whereas in Pavlov's experiment the learned response toward an initially neutral stimulus (i.e., salivation upon sound of the metronome) would typically consist of the automatic response toward an evolutionary relevant stimulus (i.e., salivation during food presentation; Unconditioned Stimulus or US). Second, the emitted response would lead to the cancelation of the impending event, making the (non-)presentation of the aversive stimulus dependent on the organism's response (Herrnstein, 1969). This procedural aspect is at odds with the standard Pavlovian procedure in which the presentation of food, or of any other US, would not depend on the animal's response (i.e., food would be presented independently of whether dogs salivated or not). Those procedural differences pointed to the potential operation of instrumental processes during avoidance learning, since in instrumental learning procedures an experimenter-defined action of the organism is necessary for outcome presentation or omission (Rescorla and Solomon, 1967). The potential involvement of instrumental processes, however, raised the question as to how avoidance responses are reinforced. Although one might intuitively argue that the source of reinforcement is the omission of the impending aversive event (i.e., the dogs flex their legs because this cancels the shock), assigning the cause of behavior to an event that has not yet occurred (i.e., shock administration) violated the dominant scientific principles of psychology at the time (i.e., the behaviorist paradigm; Watson, 1913).

A solution to that conundrum was offered in the *two-factor theory* formulated by Orval (Mowrer, 1951), who proposed that the performed response was reinforced by fear reduction (Hull, 1943). Specifically, Mowrer argued that as a result of Pavlovian fear conditioning (first factor), i.e., an antecedent stimulus (e.g., a tone) being associated with the administration of an aversive event (e.g., a shock), presentation of the antecedent stimulus will come to evoke fear. Subsequently, during the instrumental phase (second factor), escape responses that are emitted in the presence of the antecedent stimulus will be negatively reinforced by fear reduction, due to increased distance to or cessation of the antecedent stimulus. This idea was heavily inspired by the avoidance learning procedures used at the time, where avoidance responses led to the termination of the antecedent stimulus by locomotion (e.g., moving away from a shock area of a box) or by the antecedent stimulus being turned off. Of note, according to Mowrer, the omission of the aversive outcome event was to be regarded as a mere by-product of the performed CS escape behavior (Schöenfeld, 1950; Mowrer, 1960).

Two-factor theory quickly gained popularity in experimental psychology. By resorting to the concept of fear reduction during the instrumental phase, Mowrer's proposition was in line with the dominant drive reduction theories of the time (e.g., the drive reduction theory of Hull, 1943). Concurrently, by suggesting that the initial fear learning was based on Pavlovian processes, rather than drive reduction, the theory was better applicable to experimental data than the competing theory of Neal Miller (Miller, 1948), according to which all reinforcement during escape/avoidance learning originated from fear reduction.

Mowrer's theory has been used not only for explaining how maladaptive avoidance is acquired (Levis, 1981), but also as a basis for clinical interventions (Eysenck and Rachman, 1965). For example, in exposure therapy a patient is repeatedly confronted with a fearful situation or stimulus, in order to reduce that fear. It is commonly suggested that patients should be kept in the exposure situation until fear or anxiety levels have declined. This suggestion is rooted in two-factor theory and the notion that if the exposure session is terminated while fear levels remain high, the fear reduction caused by the termination of the session could promote escape or avoidance of similar situations in the future (Mathews et al., 1981; Emmelkamp, 1982; see next section for arguments against this notion).

Despite its wide influence on basic research and clinical science, two-factor theory had trouble explaining later data (see Rescorla and Solomon, 1967; Herrnstein, 1969; McAllister and McAllister, 1991, for extended discussions of two-factor theory). We now turn to some of the key criticisms against the two-factor theory.

### Criticisms against the two-factor theory

One of the strongest criticisms against the two-factor theory concerned the purported role of fear in motivating the emission of a learned escape/avoidance response. According to Mowrer's proposition, escape/avoidance is motivated by high fear levels. This notion implies that no such actions should be performed in the absence of fear. One of the first experiments to show that this may not be true was done by Solomon et al. (1953). In their experiment, dogs were first trained to jump across a barrier in response to the sounding of a buzzer previously paired with shock. The dogs then received a fear extinction treatment in which the buzzer was repeatedly presented without shock. Such extinction procedure typically leads to the reduction of fear levels (Hermans et al., 2006). If, as assumed by Mowrer, it is fear that motivates escape/avoidance, it would be expected that following Pavlovian fear extinction, dogs would also stop performing the avoidance response. The results contradicted this hypothesis: Dogs continued to jump upon sounding of the buzzer, even when the shock device had long been turned off permanently.

The observation that fear may not be necessary for avoidance has clear clinical implications. As mentioned earlier, patients are typically prevented from prematurely terminating exposure out of concern that the fear reduction resulting from termination of the session could otherwise serve as negative reinforcement for escape (Eysenck and Rachman, 1965). Experimental data, however, indicate that patients undergoing exposure therapy show similar clinical improvement regardless of whether they ended exposure while fear levels were high or low (De Silva and Rachman, 1984; Rachman et al., 1986). Taken together, both experimental data and clinical findings suggest that fear may sometimes, but not always, be involved in the maintenance of avoidance, and as such, fear and avoidance may not always “synchronize” with each other (Rachman and Hodgson, 1974).

Two-factor theory also had trouble explaining how avoidance can be acquired in the absence of an explicit antecedent stimulus. Specifically, in *unsignaled* avoidance procedures (Sidman, 1953a, 1962; see Lázaro-Muñoz et al., 2010; McCue et al., 2014 for more recent examples), rats learn to avoid shocks presented at fixed time intervals, in the absence of a discrete antecedent stimulus (Sidman, 1953a,b; see Hassoulas et al., 2013, for examples in humans). In its initial form, two-factor theory assumed the operation of explicit antecedent stimuli during the Pavlovian and the instrumental phases, stimuli that during unsignaled procedures appear to be absent.

A potential explanation for the observation of unsignaled avoidance procedures is that although not explicit, warning stimuli may still be present in a “silent” form. Temporal and proprioceptive stimuli (e.g., the passage of time), for example, could be associated with the aversive outcome (Schöenfeld, 1950; Dinsmoor, 1977). Subsequently, those stimuli could signal the presentation of an aversive event (for an alternative account centering on the role of US omission during avoidance learning see Herrnstein, 1969).

By assuming that avoidance is based on reinforcement learning, the two-factor theory also failed to explain how avoidance is acquired in naturalistic settings, where the first encounter of an organism with danger could prove fatal (Bolles, 1970; Osada et al., 2014). Similarly, when it comes to maladaptive avoidance, patients do not always report a direct traumatic event as the source of their symptomatology (Rachman, 1990). An explanation for those observations is that avoidance need not always be acquired through direct experience but can be acquired via other pathways as well (Rachman, 1991, 1977; Olsson and Phelps, 2004, 2007). Those pathways include vicarious learning (e.g., learning to be afraid of dogs after observing someone being afraid of a dog) and instructional learning (e.g., learning to be afraid of a dog after someone suggesting that dogs often attack people; Bandura and Rosenthal, 1966; Rachman, 1977). Recent evidence shows that avoidance learning can be achieved even more indirectly, such as through *symbolic generalization*. One demonstration of that was presented by Augustson and Dougher (1997). They first trained individuals to categorize eight different stimuli (i.e., A1, B1, C1, D1, A2, B2, C2, and D2) into two arbitrary categories (i.e., 1 and 2), using standard conditional discrimination procedures (Sidman, 1987): Participants first saw a target stimulus (e.g., A1) and were then asked to choose one from three stimuli presented on screen. One of those stimuli was arbitrarily assigned to the same category (e.g., B1), one stimulus to the other category (e.g., C2) and another one was irrelevant (e.g., X). Participants' task was to learn to choose the stimulus from the same category as the target stimulus (e.g., B1). In case of a correct response, the word “correct” was presented and in case of an incorrect response, the word “wrong” was presented. For example, when stimulus A1 was presented, selecting the stimulus that belonged to the same category (e.g., B1, C1 or D1) was positively reinforced whereas the selection of any other stimulus (e.g., B2 or X) was punished. Such a procedure typically results in people learning that the stimuli within each category are functionally equivalent. During a subsequent fear conditioning phase, B1 was paired with shock and B2 with the absence of shock. An instrumental phase followed, where participants could avoid shocks with a button press. Critically, results showed that, in addition to performing more avoidance responses in the presence of B1 than B2, participants also performed more avoidance responses to (unreinforced) presentations of C1 and D1 (the stimuli arbitrarily related to B1) than in the presence of C2 and D2 (the stimuli related to B2). Apparently, avoidance responses to B1 symbolically generalized to C1 and D1, which had been previously trained as equivalent to B1, without an avoidance schedule being trained for those stimuli. Those findings have recently been replicated and extended (Dymond and Roche, 2009; Dymond et al., 2011). Taken together, direct traumatic experiences may not always be necessary for the acquisition of avoidance, a proposition that is in line with contemporary views on fear learning and psychopathology (Mineka and Zinbarg, 2006).

Another argument against the two-factor theory regards the extent to which Pavlovian and instrumental learning are both necessary for the acquisition of avoidance. As a result of Pavlovian fear conditioning, a previously neutral cue will come to evoke various fear responses (e.g., enhanced physiological arousal; Beckers et al., 2013). According to emotion theories, action tendencies are an essential component of emotions (Lang, 1985; Frijda, 1988). As such, “to fear is to want to avoid.” Therefore, it could be assumed that the tendency to avoid a stimulus or situation may be acquired via purely Pavlovian learning, without instrumental reinforcement. We have recently tested this assumption. Following a differential fear conditioning procedure, during which pictures of one geometrical object were always followed by shock (CS^+^) whereas pictures of another object were never followed by shock (CS^−^), participants were faster to avoid the CS^+^ and approach the CS^−^ than vice versa in a symbolic approach-avoidance reaction time task (AAT; Krypotos et al., 2014b). Crucially, shock electrodes were detached from participants' hands during the AAT, and responses had no influence on the duration or presence of the CS, eliminating any instrumental basis for avoidance. Those results suggest that avoidance tendencies can be acquired via mere Pavlovian associations, in the absence of instrumental learning. Such acquisition of motor responses toward a CS in absence of instrumental learning has long been established in the appetitive domain. In auto-shaping procedures (Brown and Jenkins, 1968), for example, pairings of a visual CS with food would typically result in the animal (e.g., a rat or a pigeon) producing consumption responses toward the CS (e.g., licking or pecking, respectively), despite those responses being irrelevant for food presentation.

Lastly, recent findings suggest that avoidance can be evoked by the identification of predator related stimuli (e.g., smells) even in absence of a previous encounter with the predator. Specifically, mice (Osada et al., 2013) and deer (Osada et al., 2014) tend to avoid (or emit other defense-like behaviors) areas where the active components of predators' urine odors are presented, without any previous experience with that specific predator. An explanation for those findings is that such avoidance may be the result of “evolutionary memory” (Provenza, 1995) and as such, avoiding such stimuli (e.g., odors or blood) does not require the learning of associations between a stimulus (i.e., smell) and the aversive event. Of note, similar effects are yet to be demonstrated in humans.

To sum up, although influential, the two-factor theory of Mowrer proves unable to account for a series of experimental results and clinical observations. In response to those shortcomings of the two-factor theory, alternative theories have been proposed. One of those, with major influence in the experimental field, is the SSDR theory of Bolles (1970, 1971).

## Species-specific defense reactions

It has long been demonstrated that in Pavlovian conditioning, evolutionary relevant stimuli (e.g., spiders) are more readily associated with an aversive event (e.g., shock) than non-evolutionary relevant stimuli (e.g., flowers; Öhman and Mineka, 2001). Similarly, in the area of taste aversion, associations between tastes and induced sickness are acquired more readily than between audio-visual cues and sickness whereas audio-visual cues are associated more readily with shock than tastes are (the *Garcia effect*; Garcia et al., 1955; Davis and Riley, 2010).

An explanation for those findings is that by being wired to preferentially associative aversive events (i.e., shock and sickness) with phylogenetically relevant stimuli (i.e., spiders and tastes), an organism is better prepared to learn about likely cues for danger. Such ability would equip the organism with an evolutionary advantage for surviving potentially harmful cues or situations. An interesting question is whether individuals are similarly predisposed to associate the cancelation or termination of an aversive event with particular behavioral responses.

This seems to be the case. Rats will learn much more rapidly to avoid an aversive outcome by running—a behavior commonly used for avoiding a predator—than by moving their tail (Maatsch, 1959; Meyer et al., 1960; Theios et al., 1966; Masterson, 1970). Observations such as those inspired Bolles in his formulation of the Species-Specific Defense Reactions (SSDRs) theory. According to this theory, under a state of fear, organisms are phylogenetically predisposed to emit specific types of responses (e.g., fleeing, freezing, or fighting) rather than others. Bolles went one step further to suggest that for such responses, reinforcement learning is actually unnecessary; the organism just needs to learn that a stimulus predicts an aversive outcome for it to elicit an SSDR (Bolles, 1970, 1971).

The SSDR theory could indeed explain the fast acquisition of specific avoidance responses that served the evolutionary survival of the organism. Nonetheless, sometimes, an avoidance response has to be acquired that does not belong to an organism's SSDR repertoire (Crawford and Masterson, 1982). Rats, for example, can learn to avoid an aversive outcome by rearing on a wheel, a response that arguably does not usually serve survival purposes, although at a much slower rate than learning to avoid that same outcome by running on the wheel (Bolles and Grossen, 1969). In those cases, it is suggested that although under a state of fear SSDRs will initially be performed, those SSDRs will be punished by the occurrence of the aversive event, allowing for non-SSDRs to subsequently emerge. In other words, non-SSDRs would not (or not merely) be negatively reinforced, as Mowrer suggested, but they would arise because of SSDRs being positively punished by the presentation of the aversive event.

To sum up, Bolles provided a theory that could explain why some avoidance responses are acquired more readily than others, and why reinforcement may often be unnecessary for the acquisition of avoidance. Nonetheless, the theory has limitations. For one, fear states are on a continuum, ranging from low to high levels, making it difficult to define at which specific level the restriction of the behavioral repertoire to SSDRs occurs (Masterson and Crawford, 1982). Also, by rejecting reinforcement as a source for SSDRs, Bolles' theory makes it hard to explain findings suggesting that bar pressing is acquired faster when it leads to access to a safe place than when it merely leads to US omission (Crawford and Masterson, 1978). Thus, although SDDR theory provided answers to important avoidance learning questions, it fails to accurately define the conditions under which avoidance learning will occur and to provide a comprehensive account for all instances of avoidance acquisition.

## Informational factors in avoidance learning

The theories we have discussed so far provided functional explanations of avoidance learning, denying any role for cognitive or informational factors in the interpretation of avoidance acquisition. As such, those theories were in harmony with the dominant functional accounts of learning of the behaviorist paradigm. However, those functional accounts of learning failed to explain a series of laboratory phenomena such as that of *blocking* (i.e., impaired CS1-US acquisition if CS1 is paired with the US in compound with a CS2 that has been previously paired with the US in itself; Kamin, 1956, 1967, 1969) and *conditioned inhibition* (i.e., learned inhibitory responses toward CS1 if a CS1–CS2 compound is repeatedly presented without the US while CS2 is paired with the US when presented in itself; Rescorla, 1969). Such phenomena challenged traditional associative theories according to which CS-US contiguity is sufficient for Pavlovian acquisition (Rescorla, 1972; Miller et al., 1995).

As a result, a general shift in theories of learning has been observed, such that informational factors (e.g., outcome expectancies or stimulus surprisingness) started to be considered as potential explanations of acquired behavior (Rescorla, 1972).

One theory of learning with a lasting influence that was developed in that period, is the Rescorla-Wagner model (RWM; Rescorla and Wagner, 1972; Wagner and Rescorla, 1972). The basic premise of the RWM is that the rate of conditioning to a CS depends on whether the ensuing presentation of the US is surprising or not. If the CS did not elicit an accurate prediction of the (non-)occurrence of the US (negative or positive *prediction error*), learning about the CS occurs; if it did, no learning occurs. This model clearly deviates from earlier theories of learning in that it recognizes the role of informational factors (i.e., predictions) in conditioning, and despite justified criticisms (Miller et al., 1995), it is a model with great heuristic and predictive value (Beckers and Vervliet, 2009).

In the next section we present avoidance learning models that, like the RWM for Pavlovian learning, rely on informational factors to account for avoidance learning.

### The role of safety signals

In our examples of Pavlovian fear conditioning, we have so far discussed situations in which an antecedent stimulus signals an aversive event. It might be assumed that in such situations, knowledge is acquired about the relation between the antecedent stimulus and the aversive event only. However, if there are also signals present that predict the *absence* of an otherwise expected aversive event (i.e., safety signals), those will be learned about as well, and fear responses will be inhibited in the presence of those stimuli (conditioned inhibition, see above).

A relevant question then is whether, in addition to reducing fear levels, safety signals might also be able to inhibit avoidance behavior. This hypothesis was tested in a seminal study of Rescorla and LoLordo (1965), who showed that dogs would learn to jump a small barrier after the presentation of a tone previously associated with shock but would withhold avoidance upon the presentation of another tone previously associated with the absence of shock (see Weisman and Litner, 1969, for a replication). Those findings indicate that during avoidance learning, knowledge is acquired not only about stimuli that signal an aversive event, so that those stimuli come to elicit defensive behaviors (e.g., avoidance, escape), but also about stimuli that signal the absence of a forthcoming aversive event, with the latter stimuli inhibiting defensive behaviors.

Assigning a role to safety stimuli in avoidance learning allows to explain a series of data that previous theories could not account for. For example, it has been shown that avoidance can be acquired readily even if the CS is not terminated upon the performance of the experimenter-designated avoidance response (Soltysik et al., 1983; Avcu et al., 2014). This observation contradicts two-factor theory, according to which the termination of the antecedent event is necessary for fear reduction. A safety signal account can explain those data, by assuming that changes in contextual or internal cues upon performance of the experimenter-designated response can serve as safety signals. The reinforcing value of safety cues can also explain why avoidance is acquired faster if the avoidance response leads to a safe environment than if it does not (Crawford and Masterson, 1978; see also Morris, 1975; Kim et al., 2006, for related evidence).

Another observation partially supportive of a safety signal account is that rats prefer a box compartment in which a light is presented before the administration of an (unavoidable) shock, over a compartment in which shocks occur unannounced (Lockard, 1963). According to a safety signal account, the predictability of the aversive event makes the situation less unpleasant because the absence of the warning stimulus signals that the situation is safe (Seligman, 1968; Seligman et al., 1971), a view also in line with the RWM approach to inhibitory learning.

The safety signal account can also help to explain why exposure therapy does not necessarily lead to a reduction of avoidance. Specifically, it has been suggested that while undergoing exposure therapy, patients will try to reduce their unpleasantly high fear levels by engaging in overt or covert safety behaviors that reduce those fear levels (“within situation safety”; Wells et al., 1996; Salkovskis et al., 1999). Such safety behaviors may involve the generation of safety signals to reduce fear levels. For instance, individuals who are afraid to experience a panic attack while flying, might endure traveling in an airplane as long as they can carry anxiolytics with them. Having such medication in their pocket can serve as a signal that they can avoid a potential panic attack. No matter how helpful such strategy is in reducing momentary fear levels, the engagement in safety behaviors typically preserves the dysfunctional belief that the situation is inherently dangerous. By implication, on future occasions where they cannot engage their customary safety behaviors, individuals may revert into avoidance behavior in those situations.

### The cognitive theory of Seligman and Johnson

One of the most influential theories of avoidance learning, which explicitly addressed the role of informational factors, is the cognitive theory of Seligman and Johnston (1973).

In spite of its name, the theory actually contains both a cognitive and an emotional component. The cognitive component revolves around the assumption that human and non-human animals would prefer not receiving an aversive stimulus over receiving one. The cognitive component also contains the notion that as a result of an avoidance learning schedule, humans or animals would learn to expect an aversive stimulus if an avoidance response is not performed and not to expect an aversive stimulus if an avoidance response is performed. The emotional component mainly refers to the Pavlovian fear responses that develop during an avoidance learning procedure as previously described by Mowrer and others.

By incorporating a role for expectancies in avoidance learning and maintenance, the cognitive model could account for data not easily explained by two-factor theory. For example, in the experiment of Solomon et al. (1953) mentioned previously, dogs would keep jumping a barrier subsequent to a buzzer presentation previously associated with shock, even when the experimenter stopped shock administration. Since shocks no longer followed buzzer presentation, it would be expected that fear would extinguish (see Pavlovian extinction). According to two-factor theory, fear motivates escape/avoidance responses, and since fear was assumed to have extinguished, it should be expected that avoidance would diminish as well, a hypothesis that was at odds with the data. Those data, however, can be explained by the cognitive theory. According to it, avoidance is maintained despite the potential reduction in fear levels because by jumping the barrier, the animal does not directly experience that the antecedent stimulus is not followed by the aversive outcome. As such, the expectancy that an aversive event would occur after the presentation of the antecedent stimulus if an avoidance response were not emitted, is preserved.

Disconfirmation of expectancies, on the other hand, could explain why avoidance diminishes when exposure is combined with *response prevention* (Baum, 1966, 1970). During such schedule, individuals encounter a phobic stimulus while the execution of all escape responses is blocked. As a result, avoidance responses are typically thwarted when the individual encounters the antecedent stimulus in the future. This reduction in avoidance response execution, not achieved by traditional exposure programs, can be explained by considering that by not executing the escape response during the antecedent stimulus presentation, patients can realize that the expected noxious event was not to occur anyway. This then, removes the need to execute any defensive reaction during future encounters with the antecedent stimulus.

The cognitive model is not without limitations, some of which Seligman and Johnson noted themselves (Seligman and Johnston, 1973, pp. 100–101). One limitation is that the theory implicitly treats all types of avoidance responses as equivalent. Research on SSDRs suggests that this notion is untenable. Also, as noted by Lovibond (2006), the model remains silent as to how fear and expectancies relate to each other^1^. As a result, it cannot explain experimental observations such as the return of extinguished fear when the performance of the avoidance response is prevented (Solomon et al., 1953).

### Negative occasion setter account

Informational factors also play an important role in the avoidance learning theory of De Houwer and colleagues (De Houwer et al., 2005; Declercq and De Houwer, 2008). According to those authors, an avoidance response serves as a signal that a CS is not going to be followed by an aversive event, which in associative learning language is called a “negative occasion setter” (see Holland, 1992; Schmajuk and Holland, 1998, for reviews on occasion setting).

Functionally, in negative occasion setting experiments, an antecedent stimulus (e.g., a sound; CS) is followed by an outcome (e.g., a shock; US) unless another stimulus X is presented (the occasion setter, OS; e.g., a light). Accordingly, the antecedent stimulus is going to be followed by an outcome only when the occasion setter is absent and vice versa. Stimulus X thus helps to disambiguate the relationship between the antecedent stimulus and the forthcoming outcome.

Negative occasion setting can be translated to clinical situations. It could be argued, for example, that the engagement in safety behaviors, such as avoidance, serves as a signal that a specific phobic stimulus or dreaded situation is not going to result an aversive event. Going back to our earlier example, the *administration* of an anxiolytic pill could signal that being in an airplane is not going to be accompanied by a panic attack. Such a proposition is different from the safety signal account. According to this latter account, a specific stimulus is supposed to predict the absence of an aversive event directly. According to the negative occasion setter account, however, the presence of a specific event (e.g., avoidance) predicts that the otherwise valid relation between a threatening stimulus and an aversive event does not hold.

De Houwer and Declercq tested whether an avoidance response functions as a negative occasion setter by comparing the properties of avoidance behavior to properties of negative occasion setters identified in the Pavlovian literature (Holland, 1992; Schmajuk and Holland, 1998). Those properties are (a) *modulation* (i.e., CRs toward the CS are stronger in the absence than in the presence of the OS), (b) *resistance to counter conditioning* (i.e., CRs toward the CS are attenuated by the OS even if the OS has been paired with the US itself), and (c) *selective transfer* (i.e., the OS will modulate responding to other CSs that have be subject to modulation before; Holland, 1992). The experiment of De Houwer et al. (2005) was at follows.

In the first phase of their experiment, stimuli A and B were always followed by a US (shock) whereas a third stimulus C was followed by a US 50% of the times. In the second phase, the US could be prevented by pressing key X when the A stimulus was present and by pressing key Y when the B stimulus was present. The third phase consisted of the presentation of all the events occurring in the previous phases, in addition to trials in which the US occurred upon pressing the X key (X-US trials). In the crucial test phase, individuals were presented with all possible stimulus combinations, without the US ever occurring, and they were asked to rate their expectancy of a US.

Results from the test phase confirmed that avoidance responses exhibit the properties of negative occasion setters. First, participants reported higher US expectancies when the avoidance response was not available (i.e., A and B test trials) than when it was (i.e., AX and BY trials; modulation). This result shows that the function of the antecedent stimulus as a predictor of a negative outcome is dependent on the availability of the avoidance response, a finding at odds with two-factor theory but in line with other theories, such as Seligman and Johnson's cognitive model. Second, modulation was not affected by trials in which avoidance responses were followed by the US (i.e., the X-US trials; resistance to counterconditioning). This finding is in partial contrast with both the safety signal hypothesis and the cognitive model, given that the avoidance response has now become a predictor of the aversive event rather than of its omission. Third, participants would generalize this trained modulation to new stimuli, particularly those stimuli that had been involved in avoidance training (i.e., higher modulation for the AY and BX trials than for the CX or CY trials; selective transfer). Other models cannot easily account for this selectivity in trained modulation.

Despite subsequent replication and extension of those findings (Declercq and De Houwer, 2008), more recent evidence (e.g., Declercq and De Houwer, 2011) argues against the negative occasion setting account, as the property of selective transfer could also be explained by the lesser reinforcement of C compared to A and B during the first phase of the experiment. Still, despite this limitation, insights from the negative occasion setting account could prove clinically important. Current therapeutic techniques mainly target the relation between the avoidance response and the omission of an unpleasant event. The negative occasion setting account assumes that there is a hierarchical structure in avoidance learning, with the avoidance response disambiguating the relation between the antecedent stimulus and the omission, or presentation, of the aversive event (see Declercq and De Houwer, 2009, for relevant evidence). If that is the case, interventions should perhaps focus more on challenging patients' beliefs about whether their avoidance response leads to the omission of an otherwise to-be-expected noxious outcome in the presence of some antecedent stimulus, rather than on their beliefs as to whether the antecedent stimulus is a reliable predictor of the noxious event (Declercq and De Houwer, 2009).

### Lovibond's expectancy model

The most recent avoidance learning theory to include informational factors is the expectancy model of Lovibond (2006), which is basically an extension of the cognitive model of Seligman and Johnston (1973).

The expectancy model agrees with the idea that avoidance is acquired by a combination of Pavlovian and instrumental learning processes. It also accepts Seligman and Johnson's notion that during the instrumental phase, knowledge is acquired about the effects of avoidance (e.g., the *omission* of an expected unpleasant event) as well as non-avoidance (i.e., the *presentation* of an expected unpleasant event). Lastly, it aligns with the safety signal account in that it accepts that during the presentation of safety stimuli avoidance behavior is inhibited. However, the expectancy model rejects the notion that safety signals serve as positive reinforcers of the emitted response.

A major deviation of the expectancy model from the aforementioned theories is the assumption that both Pavlovian and instrumental learning are based on propositional knowledge. According to the propositional learning account, *all* learning reflects higher-order, reason-based processes, whereas earlier associative learning theories, explicitly or implicitly, rely on automatic association formation as the mechanism of learning. As such, the expectancy model assumes that avoidance learning is achieved by the accumulation of explicit knowledge about all the stimulus contingencies (e.g., that a CS is followed by a US during a Pavlovian phase) involved in avoidance learning protocols. In the same vein, the expectancy account assumes that expectancies play a crucial role not only in instrumental learning (as hypothesized by Seligman and Johnson) but in Pavlovian conditioning as well.

Most of the data we have presented so far can be accommodated by the expectancy model. Similarly to the model of Seligman and Johnston (1973), it can explain how avoidance is acquired, how it can be maintained despite Pavlovian extinction, and why response prevention during extinction will reduce avoidance. In addition, by reserving a central role for expectancies in Pavlovian learning, the expectancy model can better account for the relation between fear and avoidance. The expectancy model can explain, for example, why fear returns during response prevention, because in the absence of the avoidance response the aversive event is to be expected, and outcome expectancy is what generates fear.

Lastly, although not explicitly mentioned by Lovibond (2006), the fact that avoidance relies on propositional knowledge also allows for the acquisition of avoidance via pathways other than direct experience, rendering the account able to accommodate observations of avoidance acquired through observation, instruction, and symbolic generalization (see above).

In summary, Lovibond's expectancy model is a hybrid of earlier theories that accounts for the majority of experimental findings. Still, the model does not readily explain why some avoidance responses are learned more readily than other. It also cannot account for data in which escape/avoidance responses are acquired and expressed in the absence of instrumental reinforcement (Krypotos et al., 2014b). Lastly, it is debatable whether all elements of the expectancy model, and specifically the notion that propositional knowledge is a prerequisite for learning, could be generalized to non-human animals (Castro and Wasserman, 2009; but see Beckers et al., 2006).

## Principles of avoidance learning

We have reviewed early and more recent theories of avoidance learning, describing the strengths and limitations of each theoretical account. In this section we synthesize the strongest points of each theoretical account, distilling a set of principles of avoidance learning that collectively can account for the majority of existing data. We then consider recent experimental findings on the acquisition of avoidance tendencies in the light of those principles and discuss their potential clinical implications.

For illustration, we will describe the various phases of avoidance learning by reference to a modified version of the procedure used in the third experiment of Rescorla and LoLordo (1965) that we have briefly described above (see the section on safety signals). We use this experiment because it resembles a typical active avoidance learning procedure while also illustrating the potential inhibition of avoidance responses by safety stimuli.

In the experiment, dogs were placed in a large two-compartment box, separated by a small barrier. The experimental procedure included the presentation of a short tone (threat signal; CS^+^) always followed by shock administration (US) and the presentation of a long tone (safety signal; CS^−^) never followed by shock administration. Dogs could avoid shock administration by jumping across the barrier (i.e., the avoidance response) during the presentation of the CS^+^. As described, such procedure would typically lead to reliable execution of the avoidance response during the CS^+^ and absence of such responses during the CS^−^. We now turn to a step-by-step description of how those acquired responses can be theoretically explained.

In line with most of the theoretical accounts we have presented above, we propose that avoidance learning involves a Pavlovian component. Specifically, we assume that following a Pavlovian conditioning procedure, the presentation of the CS^+^ will elicit the expectancy of an aversive outcome, whereas the CS^−^ will elicit the expectancy of absence of the aversive outcome. As a result of those expectancies, fear responses will be evoked (e.g., physiological arousal, avoidance tendencies) in the presence of the CS^+^, whereas responses of relief (e.g., physiological relaxation, approach tendencies) are expressed in the presence of the CS^−^.

In extension to the models we have presented above, we maintain that the described Pavlovian component is sufficient for evoking avoidance tendencies. As such, we essentially treat avoidance tendencies as conditioned responses, similar to physiological arousal or subjective apprehension, elicited by the presentation of a threat cue. Such an explanation fits the data of Krypotos et al. (2014b) where avoidance tendencies were expressed in absence of any instrumental reinforcement. Going back to our example, we propose that dogs will acquire a tendency to exhibit avoidance in the presence of the CS^+^ sound prior to any instrumental learning.

Although acquired, we propose that those avoidance tendencies need not be translated into overt behavior (Strack and Deutsch, 2004). At the same time, we argue that if expressed, they will take the form of an SSDR (Bolles, 1970, 1971). In other words, the dogs in our example are expected to start jumping across the barrier upon presentation of the CS^+^, prior to the operation of any instrumental processes. Such an idea might explain the rapid learning of specific types of responses that serve avoidance, relative to other responses (Bolles and Grossen, 1969). In such cases, however, it would be more accurate to classify those responses as escape responses, expressed in a reflex-like manner, as their primary aim is to distance the organism from the CS^+^ (i.e., CS *escape*).

Despite the rapid acquisition of SSDRs by mere Pavlovian learning, the maintenance of those SSDRs, or the learning of non-SSDRs, will in fact depend on instrumental learning. As such, we adhere to the spirit of Mowrer's two-factor theory and more recent reformulations in assuming that avoidance learning depends on both Pavlovian and instrumental processes. Yet, we propose that instrumental learning plays a different role depending on whether the to-be-learned avoidance response belongs to an organism's SSDR repertoire or not. Revisiting our example, dogs are expected to start jumping across the barrier upon presentation of the CS^+^, with such response being maintained if the US stops being administrated (i.e., negative reinforcement). If, however, such a response does not lead to cancelation of the US, that particular avoidance response will cease to be performed. For illustration, assume that the experimenter-designated response changes from jumping (i.e., an SSDR) to rearing (i.e., a non-SSDR). As jumping does not result in omission of the US, another SSDR response will be performed (e.g., running within the same compartment). However, since that novel response is followed by US presentation as well, the dog will cease that response too. Eventually, when all SSDRs have been aborted, the dog may incidentally perform a non-SSDR (i.e., rearing). This response is then hypothesized to be maintained through negative reinforcement by US omission. Although such treatment of non-SSDRs bears similarities with Bolles' (1971) theory, in the sense that non-SSDRs are supposed to emerge after SSDRs are no longer performed, it also deviates from SSDR theory in that it accepts a role for reinforcement learning in the maintenance of SSDRs and the learning of non-SSDRs.

In contrast to existing models, we maintain that for the instrumental component of avoidance learning, there are multiple sources of reinforcement. Those sources include (1) the non-occurrence of an expected aversive event after performance of the avoidance response; (2) the reduction of fear due to escape from the CS^+^, at least in the initial stages of reinforcement learning; (3) the occurrence of discrete stimuli upon performance of an avoidance response that become safety signals. Although this is a bottom-up suggestion, we believe that by assuming multiple sources of reinforcement, the described principles fit a larger amount of data.

In line with both the cognitive and the expectancy model, we propose that during instrumental learning, knowledge is acquired that (1) the performance of an avoidance response in the presence of the antecedent stimulus leads to the omission of the otherwise expected aversive event and; (2) the non-performance of an avoidance response in the presence of the antecedent stimulus leads to the administration of the aversive event. Those two theoretical accounts also assume that an individual or an animal will prefer not encountering an aversive event (here a shock) over encountering one.

Lastly, we take no position here in the debate as to whether avoidance learning (and in particular the Pavlovian component thereof) is based exclusively on propositional knowledge or also relies on non-propositional association formation processes. The recent literature provides strong evidence to support a single-process propositional account of learning (see De Houwer, 2009; Mitchell et al., 2009) as well as intriguing evidence to the contrary (e.g., Sevenster et al., 2014).

### Clinical implications

The set of principles described above can help to detect sources of maladaptive avoidance. By building on those principles, clinical interventions for maladaptive avoidance can also be improved.

For instance, given that avoidance learning is assumed to depend on multiple pathways, the causes of maladaptive behavior should not be assumed to be limited to direct experience with threat. Maladaptive avoidance may just as well be the result of instructions (e.g., kids being instructed to stay away from dogs leading to a phobia toward dogs) or vicarious learning (e.g., watching a plane crash on television as an onset of avoidance of flying). Another potential pathway of avoidance learning concerns symbolic generalization (e.g., avoidance of thoughts of contamination that only symbolically relate to actual contamination; Dymond et al., 2012).

Independent of the pathway, however, patients will often be able to articulate purported relations between the antecedent stimuli and the presentation or omission of an aversive outcome, as well as the relationship between the cancelation of that aversive outcome and the performance of an avoidance response (Beck et al., 2005). Given that those relationships often reflect misconceptions of the patient (e.g., that social interactions will always end in embarrassment), those beliefs should be challenged and modified during clinical interventions, a suggestion in line with current cognitive-behavior therapy protocols.

In terms of interventions, a common therapeutic technique for anxiety disorders and phobias is exposure therapy, which entails confronting the individual repeatedly with the phobic stimulus. Clinical results suggest that exposure is necessary, but not sufficient, for the reduction of avoidance. A more effective technique for diminishing avoidance is the combination of exposure therapy with response prevention. This is consistent with existing studies that show that in order to extinguish an avoidance behavior it is not enough to omit the aversive outcome after an antecedent stimulus, it is imperative that subjects are meanwhile not allowed to perform the avoidance response (see Solomon and Wynne, 1953; Brush, 1957; Seligman and Campbell, 1965). This suggestion is based on experimental findings showing that participants engaging in avoidance and other safety-like behaviors during extinction will keep avoiding the fear conditioned stimulus when avoidance is again allowed (see the “protection-from-extinction” phenomenon; e.g., Lovibond et al., 2009). Similarly, in clinical cases, patients with social phobia will often endure an exposure session by performing subtle behaviors that reduce the level of experienced fear (“within-situation safety”; Wells et al., 1996). Helpful as that may be for enduring the phobic situation, it does not help in the modification of the initial fear beliefs (Salkovskis et al., 1999). Therefore, the combination of exposure therapy with response prevention could lead to stronger symptom reduction, despite causing sustained fear levels during exposure (Abramowitz, 1996; Neziroglu et al., 2011). In that direction, and in line with the expectancy model, a change in irrational beliefs of the clients, as typically done in cognitive-behavior therapies, could also prove potentially helpful (Whittal et al., 2005).

Finally, we have shown that avoidance tendencies can be established by mere Pavlovian association, without any instrumental reinforcement. Those tendencies are prone to return after Pavlovian extinction (Krypotos et al., 2014b). Given that such avoidance tendencies may act as precursors of overt avoidance, it might be helpful if therapeutic protocols also target the modification of action tendencies in addition to overt behavior. Encouraging results in that direction come from experimental studies on the modification of action tendencies using approach-avoidance training tasks (*training AATs*; see Wiers et al., 2010; Wittekind et al., 2015). In those tasks, participants have to primarily approach one type of stimulus and avoid another type of stimulus by using a joystick or keyboard buttons. Following such procedures, participants typically exhibit stronger approach tendencies toward the former type of stimuli and stronger avoidance tendencies toward the latter type of stimuli than before. Training AATs seem to influence corresponding overt behavior as well, at least in non-clinical populations (Taylor and Amir, 2012; Amir et al., 2013). However, translation of those findings to clinical populations has proven unsuccessful so far (e.g., Asnaani et al., 2014; Krypotos et al., in press; van Uijen et al., 2015). More research is clearly warranted here.

## Current challenges and future directions

The renewed interest in avoidance learning is apparent not only in experimental psychology research, but also in clinical psychology, psychiatry, and behavioral neuroscience (see above). With the increased interest in avoidance learning in clinical science and behavioral neuroscience come new challenges, such as a need for better communication between basic researchers and clinicians and integration of insights from neuroscience in psychological theories of avoidance learning.

### Enhancing the communication between basic scientists and clinicians

A first step for enhancing communication between basic scientists and clinicians (clinical psychologists and psychiatrists) is convergence on a common language. As an illustration of the discrepancy between the experimental and clinical use of similar terms we refer to the definition of active avoidance as used in DSM-5 (American Psychiatric Association, 2013). There it is stated that “*Active avoidance* means the individual intentionally behaves in ways that are designed to prevent or minimize contact with phobic objects or situations (e.g., takes tunnels instead of bridges on daily commute to work for fear of heights; avoids entering a dark room for fear of spiders; avoids accepting a job in a locale where a phobic stimulus is more common).” This definition does not distinguish between active and passive avoidance or between escape and avoidance; yet those behaviors might reflect the operation of distinct psychological mechanisms (see above). We believe that a common definition of different expressions of avoidance and the conditions under which it occurs, may enable clearer communication between experimental and clinical science and enhance the translation of experimental findings to clinical interventions and back.

Throughout our review, we have attempted to translate basic experimental findings to clinical situations. It is equally important that clinical observations feed into basic research. A good example for that is a recent study in which avoidance behavior led to US cancelation on some, rather than all, trials. This experimental protocol may map better on real life situations where avoidance is not always successful in preventing the occurrence of an unpleasant event (Vervliet, 2014). This should therefore provide better ecological validity to experimental findings (Vervliet and Raes, 2013).

The avoidance learning procedures we have described so far mainly focus on how someone learns to avoid an expected aversive event. This is, however, quite different from what is mostly of interest in clinical practice. Take for example someone with symptoms of spider phobia. In that case, the spider could serve as the antecedent stimulus of the aversive event, such as a venomous bite. In experimental settings, that would entail that the individual would escape the spider and avoid the bite. However, it is quite common for an individual to try and avoid the spider altogether, for example by keeping away from places where spiders are commonly found (e.g., forests). Therefore, experimental studies should develop protocols in which it is possible for someone to avoid the antecedent stimulus rather than the aversive event. Procedures that include sequential CSs (e.g., CS1 → CS2 → US relationships) could prove useful in this regard (e.g., Levis and Boyd, 1979; Malloy and Levis, 1988).

### Future developments

The theories of avoidance learning reviewed above are utterly silent with regard to the role of individual differences factors. Likewise, most experimental studies focus on how avoidance responses are acquired in general, without attending to differences between participants. However, investigating how individual differences in trait characteristics or biological factors (e.g., sex hormones) affect avoidance learning could be important for theoretical and clinical reasons.

Theoretically, the consideration of differences in biological factors or trait characteristics may help in predicting distinct avoidance learning patterns. With respect to biological factors, for example, animal research has demonstrated sex and strain differences in the rate at which avoidance is acquired (Avcu et al., 2014) and extinguished (Jiao et al., 2011). Sex differences have also been documented in humans (e.g., in Sheynin et al., 2014). Regarding trait characteristics, Lommen et al. (2010) have shown that individuals with high levels of neuroticism tend to show higher levels of avoidance toward ambiguous stimuli than individuals with low levels of neuroticism. Of importance, differences in the rate of avoidance learning might be associated with differences in other learning processes. For example, behaviorally inhibited individuals exhibit stronger conditionability between a CS and a US compared to non-inhibited individuals (Allen et al., 2014).

Clinically, the investigation of individual differences could inform the development of treatments that are tailored to specific disorders. Indeed, it has been suggested that avoidance learning patterns may differ across mental disorders (Mosig et al., 2014). Support for this suggestion comes from findings demonstrating differences in conditionability (i.e., the tendency to “acquire a larger and more persistent (autonomic) differential response to an aversive CS”; Wegerer et al., 2013) between individuals with low and high levels of spider phobia (Mosig et al., 2014). It may be hypothesized that those differences could also generalize to distinct avoidance learning patterns that differ across mental disorders. Future studies might try to unravel whether distinct patterns of avoidance constitute prognostic factors for specific disorders, allowing more targeted interventions.

Another avenue for research might be the investigation of how avoidance responding turns from goal-directed to habitual (Dickinson, 1980; Wood and Neal, 2007). If we assume that persistent maladaptive avoidance behavior (e.g., in anxiety disorders) is often instrumentally reinforced, the principles described above imply that avoidance is performed in a goal-directed manner. In other words, it is assumed that avoidance is based on knowledge about the consequences of each action and the desirability of each outcome event. However, especially in the area of psychopathology, behavior is often performed in a habitual manner, that is in a more automatic/reflexive way than goal-directed actions. Specifically, in cases such as that of anxiety disorders, maladaptive avoidance is performed repetitively, over long period of times. Such overtraining of maladaptive avoidance might result in that behavior taking the form of a habit, performed in an automatic—opposed to goal-directed—manner (Wood and Neal, 2007). This distinction is important as habitual responses have been shown to be less sensitive to extinction (Yin and Knowlton, 2006). As such, it would be worthwhile both experimentally and clinically to address the factors that turn a goal-directed avoidance action into a habit. In that direction, Ilango et al. (2014) proposed that in cases of habitual avoidance in rats, active avoidance recruits and depends on the same neural structures also involved in habit formation (e.g., the striatal-nigral-striatal circuitry), suggesting that this region should be targeted in the investigation of persistent avoidance.

Another potential source of novel insight are recent computational models of avoidance learning. One such model is the actor-critic model of Maia (2010) that is strongly inspired by the two-factor theory of Mowrer but also takes the role of prediction error in learning into account. Myers et al. (2014) also recently presented a reinforcement learning computational model that could successfully predict the acquisition of avoidance behavior in Sprague-Dawley and Wistar-Kyoto rats. Lastly, we have provided a Bayesian drift-diffusion model decomposition of performance in AAT tasks (Krypotos et al., 2014a). Such models often allow a more accurate assessment of performance and a more precise investigation of the psychological mechanisms involved in avoidance learning/performance. Of importance, knowledge of the cognitive mechanisms in play during maladaptive behavior expression could serve the development of more targeted clinical interventions (Kazdin, 2008).

Over the past decade, behavioral neuroscience research has greatly expanded our insight into the neural correlates of avoidance learning. It has been shown, for example, that avoidance learning in humans correlates with activation of amygdala, which is known to play a key role in the acquisition of Pavlovian fear responses (Phelps and LeDoux, 2005), and the striatum (O'Doherty, 2004), which is involved in learning about rewards (Delgado et al., 2009). In rodent studies, areas such as the amygdala (Cain and LeDoux, 2008), the infralimbic prefrontal cortex (Moscarello and LeDoux, 2013), and the prefrontal striatal circuits (Bravo-Rivera et al., 2014; see also Phelps and LeDoux, 2005; Ilango et al., 2014 for reviews) have also been shown to play a role in avoidance acquisition. Findings such as these help to further increase our understanding of how Pavlovian and instrumental learning processes shape avoidance behavior.

Exciting possibilities for the deeper investigation of the neural correlates in avoidance acquisition open up with the introduction of new methodologies. For example, in order to mimic the behavior of natural predators, Choi and Kim (2010) used the Robogator (LEGO Mindstorms robot) while rats where approaching or avoiding food stimuli. The results showed that rats' foreaging behavior increased during amygdala inactivation and decreased during amygdala activation. Also, Bravo-Rivera et al. (2014) attempted to dissociate the neural circuits mediating active avoidance in rats. Importantly, researchers argued against the use of the traditional shuttlebox, where both compartments can predict shock making it difficult to differentiate between circuits involved in active avoidance and escape. Instead, they used a new paradigm where a US could be avoided if animals would step on a nearby platform that had never been shocked before. The results showed that active avoidance is mediated by the prefrontal-striatal circuit. Further neurobiological dissection of the differences between passive avoidance, active avoidance, and escape is likely to necessitate updating of psychological theories of avoidance learning, which typically assume that they rely on similar processes. In that respect, exciting possibilities for studying the neural circuits of avoidance acquisition open up with the use of optogenetics (see Kravitz et al., 2012; Nabavi et al., 2014; Namburi et al., 2015 for recent examples).

## Conclusions

Research on avoidance learning had waned since the 1970s, leaving key questions unanswered. In light of the recent renewal of interest in avoidance in behavioral and brain research and in clinical science, we have provided a review of the most prominent historical and modern avoidance learning theories and relevant empirical findings. This review has yielded a number of essential principles of avoidance learning that should be useful for experimental researchers as well as clinicians.

Many questions remain to be answered. We have highlighted topics for future research as well as ways to enhance communication between basic and clinical science. By doing so, we hope that the present paper contributes to further research on the psychological and biological basis of avoidance and helps to pave the way for novel interventions.

## Author contributions

AMK and TB wrote the manuscript. MK and ME provided critical feedback to the manuscript. All authors have approved the final version of the manuscript.

## Conflict of interest statement

The authors declare that the research was conducted in the absence of any commercial or financial relationships that could be construed as a potential conflict of interest.

## Footnotes

^1^Notably, and in contrast to more recent models of avoidance learning (e.g., the expectancy model; Lovibond, 2006), Seligman and Johnston (1973) did not assign a mediating role to expectancies for Pavlovian learning, although they did not fully exclude such possibility either (see footnote 14 in Seligman and Johnston, 1973).
